# Wall-L merge sort: A tunable and adaptive sorting algorithm for diverse computing environments

**DOI:** 10.1371/journal.pone.0341993

**Published:** 2026-02-02

**Authors:** Mohammad Abdur Rob, Md. Zakir Hossen, Md. Kamal Hossen, Md. Mithun Ali, Bhaskor Roy

**Affiliations:** Department of Computer Science and Engineering, Dhaka University of Engineering & Technology (DUET), Gazipur, Bangladesh; Xidian University, CHINA

## Abstract

Sorting algorithms play a crucial role in computing, but most are designed with rigid structure that are only efficient under certain conditions. Although some sorting algorithms perform well in some circumstances, they do not perform well on some resistant platforms. This study introduces Wall-L Merge Sort, which combines quadratic sorting with a modifiable multi-layer merging approach. By setting a single parameter, *L*, which determines the number of merge layers, Wall-L Sort shows a transition in the time complexity from *O*(*n*^2^) to O(nlogn) without any modification in the unique idea. This degree of freedom enables a broad variety of input sizes to be encompassed and expands to several constraint platforms. The results show that Wall-L Sort and K-way Merge Sort have the built-in ability to handle different situations where other algorithms fail without assistance functions. Wall-L Merge Sort is the only sorting algorithm that combines complexity tuning, cache efficiency, recursion depth control, parallelism, and broad adaptability into one framework. It may not be the best choice for every situation, but its flexibility makes it a good fit for many different platforms, from small embedded systems to big computing systems. The theoretical and empirical evidence in this paper substantiates these advantages.

## 1 Introduction

Sorting is the process of organizing items in a particular sequence, such as in ascending or descending order. It is very important for tasks like matching items and searching for keys in information files, and it is often a requirement for different algorithms [1,2]. But sorting can be harder, take more time, and use more resources than searching. When you sort the data, you can search for it quickly and easily many times [3]. In real life, sorting is useful for a lot of things.

There are many sorting algorithms, which are categorized based on their time complexities: quadratic, logarithmic, and linear. Quadratic sorting algorithms have a time complexity of *O*(*n*^2^), such as bubble sort and insertion sort. Logarithmic algorithms, like quick sort and merge sort, typically have a complexity of O(nlogn). Linear-time algorithms, with a complexity of *O*(*n*), can only be achieved under specific conditions or constraints on the dataset. No single algorithm is optimal for every type of dataset; each has specific use cases where it performs best [4]. This is why developing new sorting algorithms remains a popular and important area of research.

Quadratic sorting algorithms, such as bubble sort, selection sort, and insertion sort, work well for small datasets and are easy to implement [5]. However, their main limitation is their inefficiency with larger datasets, leading to the need for improved versions. Many enhanced versions of quadratic algorithms have been developed. For example, the paper [6] introduces an improved version of bubble sort called “smart bubble sort,” which outperforms traditional bubble sort and merge sort in the best case. Furthermore, [7] presents an enhanced insertion sort with a complexity of *O*(*n*^1.585^), and [8] introduces an insertion sort that reduces time complexity by 23% compared to the traditional version. Improved selection sort algorithms have also been explored [9,10]. There are also efforts to combine algorithms for better performance. For instance, [11] presents a combined version of Bubble Sort and Insertion Sort that outperforms both traditional algorithms. All of the improved version of the quadratic sort have achieved slightly better complexity than the original, but are not adjustable to various data sizes. Moreover, non-comparison-based algorithms like Bucket Sort [12] can reduce the time complexity to *O*(*n*). However, Bucket Sort has a worst-case complexity of *O*(*n*^2^), making it unsuitable for all types of datasets.

Comparison-based sorting algorithms like Quick Sort, Merge Sort, and Heap Sort are the most commonly studied sorting techniques because they are applicable in general scenarios and theoretically come with guarantees. Heap Sort, proposed by Williams [13], ensures a time complexity of O(nlogn) for all inputs and is more predictable compared to Quick Sort. However, heap operations tend to be less practical and involve a worse constant factor, along with memory-access inefficiencies. Similarly, Merge Sort [1] guarantees a time complexity of O(nlogn) for all inputs and is widely used in external sorting due to its stable performance and predictable access patterns. However, it requires an additional *O*(*n*) auxiliary memory for merging, which makes it less suitable for memory-constrained environments.

Quick Sort, first introduced by Hoare [14], is one of the fastest comparison-based algorithms in practice. It has an average-case time complexity of O(nlogn). Yet, its worst-case complexity of *O*(*n*^2^) under poor pivot selection (e.g., sorted or nearly sorted inputs) results in it being less stable. Optimized versions, such as randomized Quick Sort, can overcome this limitation, but it still has a fixed theoretical worst-case complexity of *O*(*n*^2^). K-way merge sort is a high-performance external sorting algorithm, particularly effective for large datasets stored in external memory. Its merge depth is approximately $\log_{K}n$, which depends on both the input size and the degree of merging *K* [15].

Merge Sort, K-way merge sort, and Quick Sort are examples of logarithmic-time sorting algorithms that usually have a recursion depth of O(logn). This is fine for general-purpose systems, but recursive implementations don’t have clear stack control, which can cause stack overflow in systems with limited stack memory. In these situations, it is better to do iterative re-implementations to make sure that the stack is safe and that resources are used in a way that is easy to predict. In parallel computing environments, recursive implementations are better for scalability and expressiveness because divide-and-conquer recursion naturally fits with parallel tasks.

There are hybrid algorithms that run in *O*(*n*) in the best case and O(nlogn) in the worst case for almost sorted input sequences. For example, Timsort [16] is a hybrid sorting algorithm that combines Merge Sort and Insertion Sort. It detects and exploits existing runs in the input to optimize performance, achieving O(nlogn) complexity in the worst case. For small runs, it uses insertion sort; for larger runs, it applies a stable merge process. Timsort delivers excellent practical performance on partially ordered data. It operates iteratively using a run stack, avoiding recursion and stack overflow. However, its run-dependent scheduling introduces sequential dependencies that limit parallelism.

Introsort (Quicksort + Heapsort + Insertion Sort) is a practical hybrid sorting algorithm that employs an adaptive switching strategy based on recursion depth [17]. It begins with Quicksort and switches to Heapsort when the recursion exceeds a predefined depth(not dynamic stack control) threshold, while Insertion Sort is used for small partitions. Although efficient in general-purpose scenarios, Introsort does not dynamically adapt to cache hierarchies, parallel hardware, or other platform-specific characteristics. While several performance-optimized hybrid sorting algorithms have been proposed [18–21], they often rely on fixed thresholds for switching between sorting strategies, resulting in abrupt phase transitions and limited adaptability across diverse hardware architectures.

In some cases, when data possess specific properties, non-comparison-based sorting algorithms can break the O(nlogn) barrier. For instance, Counting Sort and Radix Sort achieve time complexities of O(n+k) (where *k* is the range of input values) and O(d ⋅ (n  +  b)) (where *d* is the number of digits and *b* is the base), respectively [22,23]. These methods are favorable for datasets with smaller ranges or fixed-length strings but are highly data-dependent and not generalizable. As per Orfao and Ruiz-Argüelles [24], the cell sorting technique takes *O*(*n*) in best-case scenarios yet can reach to *O*(*n*^2^) in advanced methods. Drawbacks include high cost, operational complexity, and susceptibility to sample quality. Non-comparison-based sorting algorithms require specific constraints on the input data and are not generalizable to all types of datasets.

The existing literature lacks a comparison-based sorting algorithm that is both predictable and theoretically aligned, with a natural scope for competitive performance across diverse computing environments ranging from high-performance systems to constrained computing platforms. In particular, there is a lack of stable algorithms with natural adaptability and survivability in stack-limited parallel environments, where recursive structures are preferable. Traditionally, algorithms are evaluated based only on time and space complexity, but it is equally important to assess their behavior under constrained computational conditions.

This paper introduces the Wall-L Merge Sort, a sorting algorithm designed with predictable complexity across diverse computing environments and a natural algorithmic scope for handling constrained situations. Wall-L achieves optimal asymptotic time complexity, predictable stack control, inherent parallelism, and efficient cache utilization, making it highly suitable for varied computing platforms. Furthermore, this paper aims to evaluate existing algorithms under different environmental constraints to identify which ones theoretically possess a natural capability to handle diverse computational conditions, alongside the proposed Wall-L Sort.

## 2 Concept of Wall-L Merge Sort

The term Wall-*L* refers to the *L*-th layer wall, where *L* represents the layer. The main concept behind this is as follows: If we initially have a dataset of length *n*, we divide this data into several blocks. The boundary of each block is referred to as a wall. When dividing the initial *n*-length data into blocks, we call these blocks Wall-*L* blocks or *L*-th layer blocks.

Now, suppose after dividing the *n*-length data into blocks, we obtain blocks with a maximum of *K*_*L*_ elements in each block of Wall-*L*. Next, we further divide each block of Wall-*L* into smaller blocks. These smaller blocks are referred to as Wall-(*L*–1) blocks or (*L*–1)-th layer blocks, and their boundaries are called (*L*–1)-th layer walls. If we get a maximum of *K*_*L*−1_ elements in each block of Wall-(*L*–1), we continue this process by dividing the *K*_*L*−1_-length data into blocks for Wall-(*L*–2), and so on, until we reach Wall-1 blocks.

Each block of Wall-1 contains a maximum of *K*_1_ elements. We then apply a quadratic time sorting algorithm to sort the elements within each Wall-1 block. After sorting, we merge all Wall-1 blocks within each separate block of Wall-2. Then, we merge all Wall-2 blocks within each separate block of Wall-3. This process continues until we merge all Wall-*L* blocks, resulting in the final sorted list.

Each block of Wall-*L* contains at most:


$$K_{L}={\left(\frac{Ln}{2}\right)}^{\frac{L}{L+1}}\;\text{elements.}$$

Each block of Wall-(*L*–1) contains at most:


$$K_{\text{L-1}}={\left(\frac{(L-1)K_{L}}{2}\right)}^{\frac{\left(L-1\right)}{L}}\;\text{elements.}$$

Each block of Wall-(*L*–2) contains at most:


$$K_{\text{L-2}}={\left(\frac{(L-2)K_{\text{L-1}}}{2}\right)}^{\frac{\left(L-2\right)}{\left(L-1\right)}}\;\text{elements.}$$

We continue this process until we reach Wall-1 blocks.

Each block of Wall-1 contains at most:


$$K_{\text{1}}={\left(\frac{K_{\text{2}}}{2}\right)}^{\frac{1}{2}}\;\text{elements.}$$

**Fig 1:**
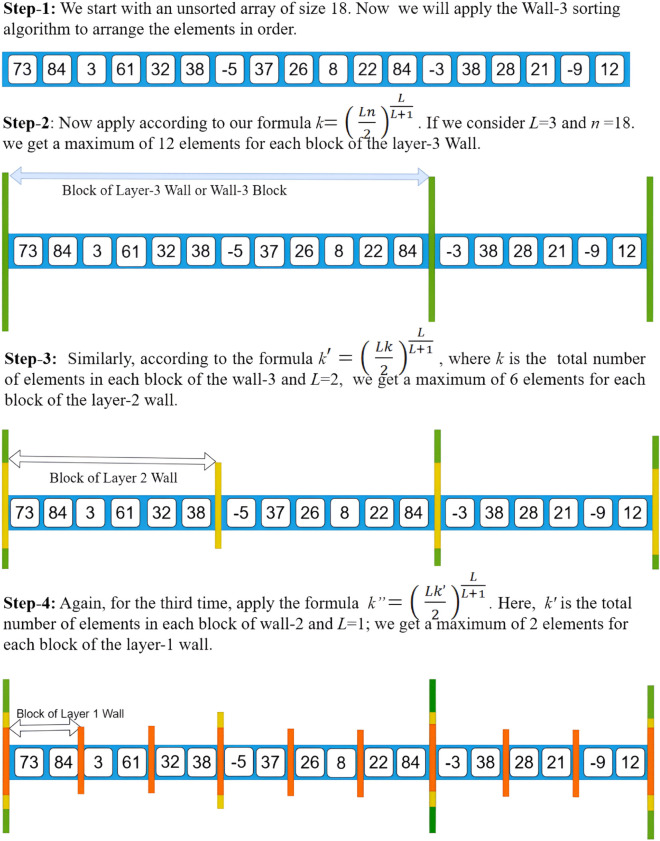
Wall-3 Sort example (first page).

**Fig 2:**
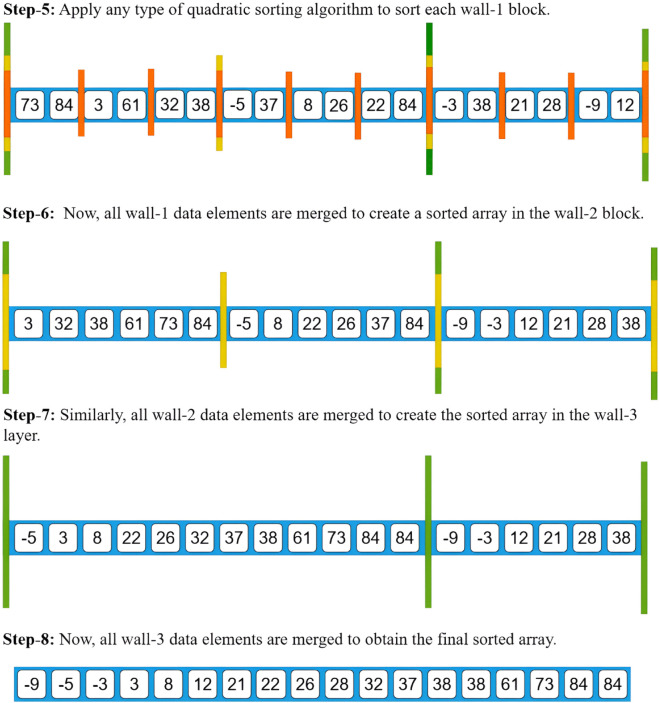
Wall-3 Sort example (second page).

This formula will be proved in the theoretical section. Using this formula, we will now present an example of the Wall-3 Merge Sort algorithm in Figs 1 and 2.

## 3 Theoretical analysis

**Wall-1 merge sort:** Suppose our data size is *n*. First, we divide the data into blocks, with each block containing *K* elements. We then apply a quadratic-time algorithm to each block of *K* elements. The time complexity of the core sort for the worst case will be:


$$TC_{\text{core sort}}=K^{2}+K^{2}+K^{2}+\cdots \text{(total of }\frac{n}{K}\text{ terms)}$$

$$=K^{2}\left(\frac{n}{K}\right)$$

=nK

Next, we calculate the time complexity required for merging the $\frac{n}{K}$ blocks. We know that if two sorted arrays have lengths *m* and *n*, the worst-case complexity for merging them is O(m  +  n) [22]. Therefore, the time complexity of the merging phase is:


$$TC_{\text{merge}}=2K+3K+4K+\cdots +\frac{n}{K}K$$

$$TC_{\text{merge}}=K(2+3+4+\cdots +\frac{n}{K})$$

$$=K\left(\frac{{\left(\frac{n}{K}\right)}^{2}+\frac{n}{K}}{2}-1\right)$$

$$=\frac{n^{2}}{2K}+\frac{n}{2}-K$$

For K≥1, since $\frac{n^{2}}{2K}+\frac{n}{2}>\frac{n^{2}}{2K}+\frac{n}{2}-K$, and if $\frac{n}{K}$ is not an integer, then the previous term of $\frac{n}{K}$ must be $\left\lfloor \frac{n}{K}\right\rfloor$.

Let,


$$m=\left\lfloor \frac{n}{K}\right\rfloor$$

Then, $\frac{n}{K}=m+q$ where 0 < *q* < 1.

So we can write,


$$\frac{m(m+1)}{2}<\frac{\left(m+q)(m+q+1\right)}{2}$$

$$\frac{m(m+1)}{2}-1<\frac{\left(m+q)(m+q+1\right)}{2}-1$$

$$(2+3+4+\cdots +m)<\left(\frac{{\left(\frac{n}{K}\right)}^{2}+\frac{n}{K}}{2}-1\right)$$

$$(2+3+4+\cdots +m+\frac{n}{K})<\left(\frac{{\left(\frac{n}{K}\right)}^{2}+\frac{n}{K}}{2}-1\right)+\frac{n}{K}\;$$

Thus, we can conclude that $\frac{n}{K}$ is integer or not,


$$K(2+3+4+\cdots +\frac{n}{K})<\frac{n^{2}}{2K}+\frac{3n}{2}$$

or


$$K(2+3+4+\cdots +\frac{n}{K})\approx \frac{n^{2}}{2K}+\frac{n}{2}$$

By the definition of Big-O notation, we can take


$$TC_{\text{merge}}=\frac{n^{2}}{2K}+\frac{n}{2}$$

Thus, the total time complexity of Wall-1 Merge Sort is:


$$TC_{\text{Wall-1 Merge}}=nK+\frac{n^{2}}{2K}+\frac{n}{2}$$

We have derived the time complexity of Wall-1 Merge Sort, but the value of *K* is yet to be determined. Our goal is to find the value of *K* that minimizes the overall time complexity. To achieve this, we use differential calculus.

Let


$$f(K)=TC_{\text{Wall-1 Merge}}=nK+\frac{n^{2}}{2K}+\frac{n}{2}$$

Now, we calculate the first derivative of *f*(*K*):


$$\frac{d}{dK}f(K)=n-\frac{n^{2}}{2K^{2}}$$

To find the minima, we set the derivative equal to zero:


$$n-\frac{n^{2}}{2K^{2}}=0$$

$$K=\pm \sqrt{\frac{n}{2}}$$

Next, we calculate the second derivative of *f*(*K*):


$$\frac{d^{2}}{dK^{2}}f(K)=\frac{2n^{2}}{K^{3}}$$

From this, it is clear that for $K=\sqrt{\frac{n}{2}}$, we have $\frac{d^{2}}{dK^{2}}f(K)>0$, meaning that $K=\sqrt{\frac{n}{2}}$ corresponds to a minimum.

Substituting $K=\sqrt{\frac{n}{2}}$ into the original equation for *f*(*K*):


$$f\left(\sqrt{\frac{n}{2}}\right)=n\sqrt{\frac{n}{2}}+\frac{n^{2}}{2\sqrt{\frac{n}{2}}}+\frac{n}{2}$$

Thus, according to Big-O notation, the overall time complexity of Wall-1 Merge Sort is:


$$TC_{\text{Wall-1 Merge}}=n\sqrt{\frac{n}{2}}+\frac{n^{2}}{2\sqrt{\frac{n}{2}}}+\frac{n}{2}$$

$$=\sqrt{2}n^{\frac{3}{2}}+\frac{n}{2}$$

Therefore, the worst-case time complexity for Wall-1 Merge Sort is O(n ⋅ $n^{\frac{1}{2}})$, where each sub-block contains $\sqrt{\frac{n}{2}}$ elements.

**Wall-2 Merge sort:** To implement Wall-2 Merge Sort, similar to Wall-1 Merge Sort, we first divide our total data into blocks, where each block contains *K* elements. Thus, we find a total of $\frac{n}{K}$ blocks. To sort each block of *K* elements, we apply the Wall-1 Merge Sort technique. As previously proved, the time complexity of Wall-1 Merge Sort is $O(n\cdot n^{\frac{1}{2}})$. Therefore, the time complexity of the core sort in Wall-2 Merge Sort will be:


$$TC_{\text{core sort}}=KK^{\frac{1}{2}}+KK^{\frac{1}{2}}+KK^{\frac{1}{2}}+\cdots \text{(total of }\frac{n}{K}\text{ terms)}$$

$$=KK^{\frac{1}{2}}\left(\frac{n}{K}\right)$$

$$=nK^{\frac{1}{2}}$$

Next, the time complexity for merging the $\frac{n}{K}$ blocks will be:


$$TC_{\text{merge}}=\frac{n^{2}}{2K}+\frac{n}{2}$$

Thus, the total time complexity of Wall-2 Merge Sort is:


$$TC_{\text{Wall-2 Merge}}=nK^{\frac{1}{2}}+\frac{n^{2}}{2K}+\frac{n}{2}$$

Now, using a similar approach as in Wall-1 Merge Sort, we apply differential calculus to find the value of *K* that minimizes the time complexity. From this, we get:


$$K=n^{\frac{2}{3}}$$

Using this value of *K*, the time complexity of Wall-2 Merge Sort becomes $O(n\cdot n^{\frac{1}{3}})$.

Similarly, by using Wall-2 Merge Sort in the core sort of Wall-3 Merge Sort, we find that the time complexity of Wall-3 Merge Sort is O(n ⋅ $n^{\frac{1}{4}})$. Furthermore, applying Wall-3 Merge Sort in the core sort of Wall-4 Merge Sort gives a time complexity of $O(n\cdot n^{\frac{1}{5}})$, and so on. Now we will try to find the general case.

**Wall-L Merge Sort:** For finding the general case . similarly we divide our total data into $\frac{n}{K}$ blocks where each blocks contain *K* elements. then we will apply Wall-(L-1) Merge sort in each block of Wall-L merge sort. Suppose the worst case time complexity of Wall-(L-1) is $O(n\cdot n^{\frac{1}{L}})$ So the time Complexity of core sort of Wall-L sort will be,


$$TC_{\text{core sort}}=KK^{\frac{1}{L}}+KK^{\frac{1}{L}}+KK^{\frac{1}{L}}+\cdots \text{(total of }\frac{n}{K}\text{ terms)}$$

$$=KK^{\frac{1}{L}}\left(\frac{n}{K}\right)$$

$$=nK^{\frac{1}{L}}$$

Again, the time complexity for merging the $\frac{n}{K}$ blocks will be:


$$TC_{\text{merge}}=\frac{n^{2}}{2K}+\frac{n}{2}$$

So, the total time complexity of Wall-L Merge Sort is:

$$TC_{\text{Wall-L Merge}}=f(K)=nK^{\frac{1}{L}}+\frac{n^{2}}{2K}+\frac{n}{2}$$ (1)

Now we will find the value of *K* using differential calculus for minima To find the minimum of the function $f(K)=nK^{\frac{1}{L}}$  +  $\frac{n^{2}}{2K}+\frac{n}{2}$. We get using differential calculus that *f*(*K*) has a minimum at $K=\left(\frac{nL}{2}\right){\;}^{\frac{L}{L+1}}$.

Now, substituting the value of *K* into Eq (??), we get:


$$TC_{\text{Wall-L Merge}}=f(K)=n{\left({\left(\frac{nL}{2}\right)}^{\frac{L}{L+1}}\right)}^{\frac{1}{L}}+\frac{n^{2}}{2{\left(\frac{nL}{2}\right)}^{\frac{L}{L+1}}}+\frac{n}{2}$$

From this, we can conclude that the time complexity of Wall-L merge sort is $O\left(n\cdot n^{\frac{1}{L+1}}\right)$. It is proven according to mathematical induction that if we apply Wall-(L-1) merge sort, which has time complexity $O\left(n\cdot n^{\frac{1}{L}}\right)$, in the core sorting step of Wall-L sort, then the time complexity of Wall-L sort will be $O\left(n\cdot n^{\frac{1}{L+1}}\right)$ [25]. Additionally, each sub-block of Wall-L sort contains


$$K={\left(\frac{nL}{2}\right)}^{\frac{L}{L+1}}\text{ elements}.$$

Now, let’s clarify how many elements each layer of Wall’s sub-blocks can contain. Suppose we have a total data size *n*; to sort them, we will apply Wall-L merge sort.

In the *L*-th layer blocks, called *L*-th layer wall’s blocks (more briefly, Wall-L blocks), each block contains a maximum of


$$K_{\text{L}}={\left(\frac{nL}{2}\right)}^{\frac{L}{L+1}}\text{ elements}.$$

Each block of the (*L*–1)-th layer’s wall, under each block of the *L*-th layer’s wall, contains a maximum of


$$K_{\text{L-1}}={\left(\frac{K_{\text{L}}(L-1)}{2}\right)}^{\frac{L-1}{L}}\text{ elements}.$$

Similarly, each block of the (*L*–2)-th layer’s wall, under each (*L*–1)-th layer’s wall, contains a maximum of


$$K_{\text{L-2}}={\left(\frac{K_{\text{L-1}}(L-2)}{2}\right)}^{\frac{L-2}{L-1}}\text{ elements}$$

Recursively, we find the number of elements each block contains according to the corresponding wall until we reach the blocks of the 1st layer’s wall. Then, we apply a quadratic-time sorting algorithm to each block of the Wall-1. After sorting each block of Wall-1, we merge each block of Wall-1, then merge each block of Wall-2, and so on, until the blocks of Wall-L are merged to form the final sorted list.

Now, let’s discuss the maximum value of *L*. We can arbitrarily choose the value of *L* within this limit, depends on the size of the data *n*. Let’s prove it.

We found that $K=\left(\frac{nL}{2}\right){\;}^{\frac{L}{L+1}}$, but the total data size is *n*. Therefore, we must have *K* < *n*. To find the maximum value of *L*, we consider *K* = *n*, then


$${\left(\frac{nL}{2}\right)}^{\frac{L}{L+1}}=n$$

which simplifies to


$$n={\left(\frac{L}{2}\right)}^{L}.$$

Thus, we have to choose *L* such that $n>\left(\frac{L}{2}\right){\;}^{L}$ in order to achieve the time complexity $O\left(n\cdot n^{\frac{1}{L+1}}\right)$.

## 4 Result and discussion

### 4.1 Complexity transition

*L* is an arbitrary constant that can be chosen such that the condition $n>\left(\frac{L}{2}\right){\;}^{L}$ is satisfied. From Table 1, we observe that if the data size n>65,536, then any *L* from the range [1,8] can be selected. Similarly, if *n* > 7^14^, then any *L* from [1,14] is acceptable.

**Table 1:** Data size condition and time complexity for different values of *L.*

*L*	$\left(\frac{L}{2}\right){\;}^{L}$	Data Size Condition $n>\left(\frac{L}{2}\right){\;}^{L}$	Time Complexity
0	0	*n* > 0	$𝒪(n^{2})$
2	1	*n* > 1	$𝒪(n^{4/3})$
4	16	*n* > 16	$𝒪(n^{6/5})$
6	729	*n* > 729	$𝒪(n^{8/7})$
7	7,529.535	n>7,529.535	$𝒪(n^{9/8})$
8	65,536	n>65,536	$𝒪(n^{10/9})$
10	9,765,625	n>9,765,625	$𝒪(n^{12/11})$
11	169,835,630.41	n>169,835,630.41	$𝒪(n^{13/12})$
13	25,166,294,529.5	n>25,166,294,529.5	$𝒪(n^{15/14})$
14	678,223,072,849	n>678,223,072,849	$𝒪(n^{16/15})$

A larger value of *L* yields better theoretical time complexity. However, since L≪n, it acts as a tunable constant rather than a mandatory changeable parameter. The value of *L* can be adjusted to optimize performance when necessary. In practice, a single well-chosen value of *L* is often sufficient to meet efficiency demands. Thus, *L* is considered a tunable constant, and the value of *L* must be an integer.

Wall-L has a significant feature: its time complexity transforms from *O*(*n*^2^) to O(nlogn) simply by tuning the parameter *L*, without changing the core algorithm. The general time complexity is given by


$$T(n)=O\left(n\cdot n^{\frac{1}{L+1}}\right).$$

If *L* = 0, then the complexity becomes $O(n\cdot n^{1})=O(n^{2})$. Now, we analyze the behavior for large values of *L*.

As predefined, *L* is a tunable constant and not a function of *n*. However, to analyze the approximate behavior for large *n*, we consider the boundary case where *L* reaches its largest allowable value. Solving the inequality $n>\left(\frac{L}{2}\right){\;}^{L}$ asymptotically gives


$$L≲\frac{\log n}{\log\log n}.$$

Substituting this upper bound into the time complexity expression:


$$T(n)=n\cdot n^{\frac{1}{L+1}}\approx n^{1+\frac{1}{L+1}}.$$

Using the approximation $L\approx \frac{\log n}{\log\log n}$, we get:


$$\frac{1}{L+1}\approx \frac{\log\log n}{\log n},\;\text{so}\;T(n)\approx n^{1+\frac{\log\log n}{\log n}}=O(n\log n).$$

Therefore, the transition of Wall-L’s time complexity lies in the range:


$$[n^{2},\;n\;\log\;n].$$

**Fig 3:**
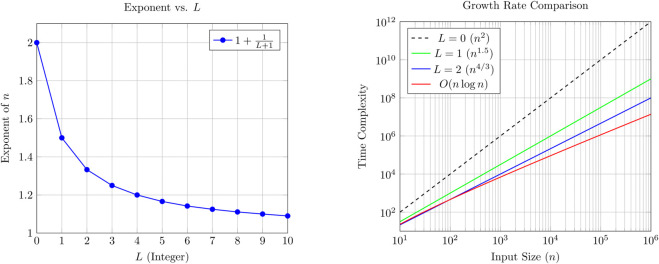
Wall-L complexity analysis: (Left) Exponent of n decreases as L increases; (Right) Actual growth rates show how higher L values approach O(nlogn) behavior.

The Fig 3 demonstrates that Wall-L allows smooth complexity control through *L*, adapting from quadratic to optimal logarithmic behavior.

### 4.2 Cache awareness and stack controlling

Merge Sort is generally considered more cache-efficient than Quick Sort and Heap Sort due to its blocking behavior and sequential access patterns during merging. Although it does not offer the most optimal cache performance, unlike algorithms such as Funnel Sort or Distribution Sort, it still exhibits relatively good cache efficiency in practice [26]. Under the I/O Cache model, which assumes a fully associative cache with optimal replacement, a cache size *Z*, and block transfer size *P*, we analyze the worst-case cache miss complexity of merge Sort [27].

In classic Merge Sort, each level of recursion incurs $\frac{n}{P}$ cache misses, and the recursion depth is $\log\frac{n}{Z}$, yielding a total cache miss cost:


$${\text{CacheMiss}}_{\text{MergeSort}}=O\left(\frac{n}{P}\log\frac{n}{Z}\right).$$

The worst-case cache miss complexity of Wall-*L* Sort is $O\left(\frac{n}{P}\cdot n^{\frac{1}{L+1}}\right)$. For the asymptotic case where $L=\frac{\log(n/Z)}{\log\log(n/Z)}$, the cache miss complexity of Wall-*L* reduces to $O\left(\frac{n}{P}\log\frac{n}{Z}\right)$, similar to Merge Sort. From Table 2, it is clearly shown that for larger values of the parameter *L*, the last-layer blocks (i.e., Wall-1 blocks) contain a relatively small number of elements, despite the large input size *n*. Hence, it is reasonable to assume that each Wall-1 block fits into cache, i.e., *k*_1_<*Z*. Also, its built in blocking strategy and upper sequential merge layers maintain better spatial locality, like Tailed Merge Sort. So Wall-*L* is also a cache-friendly algorithm.

**Table 2:** Wall-1 block sizes for different input sizes and layer depths *L.*

Input Size (*n*)	Number of Layers (*L*)	Wall-1 Block Size
65,536	8	5
9,765,625	10	9
169,835,630	11	12
25,166,294,529	13	18
678,223,072,849	14	23

If any implementation includes the use of buffering in the merging strategy (which prevents more cache misses), it can lead Wall-*L* Sort to achieve optimal cache complexity, alongside other adaptable features of Wall-*L*. Funnel Sort has optimal cache and time complexity, but it heavily depends on recursion, lacks stack-overflow protection features, and is difficult to support parallelism by nature. These factors make Funnel Sort challenging to implement in practice, particularly in environments with limited stack space or parallel processing requirements. Wall-L Sort, by comparison, offers more implementation flexibility and is better suited to modern memory hierarchies and parallel architectures by nature.

Additionally, Recursive Wall-*L* Sort offers stack-depth control, a feature absent in most other recursive algorithms. Unlike Quick Sort or Heap Sort, which have a fixed recursive depth of approximately logn, Wall-*L* Sort allows independent tuning of recursive depth regardless of input size *n*. As shown in Table 3, the recursive depth of Wall-*L* Sort can be adjusted independently of *n*, whereas other algorithms inherently depend on *n*. For example, with a data size of 169,!835,!630, traditional logarithmic-depth algorithms require approximately 28 levels of recursion. While *k*-way merge can reduce the depth by increasing *k*, it still depends on *n*. In contrast, Wall-*L* Sort can operate with any depth between 1 and 11, with its tunable parameter *L* that explicitly controls the recursion depth.

**Table 3:** Stack depth comparison: Classical log_2_*n* Algorithms vs. k-way Merge Sort and Wall-L.

Input Size *n*	Depth (log_2_*n*)	k-way Merge Sort Depth $\left\lceil \log_{k}n\right\rceil$	Wall-L Depth
65,536	16	k = 2 | 16 k = 3 | 13 k = 5 | 10	L = 1 to 8
9,765,625	24	k = 2 | 24 k = 3 | 20 k = 5 | 15	L = 1 to 10
169,835,630	28	k = 2 | 28 k = 3 | 24 k = 5 | 18	L = 1 to 11
25,166,294,529	35	k = 2 | 35 k = 3 | 31 k = 5 | 24	L = 1 to 13
678,223,072,849	40	k = 2 | 40 k = 3 | 37 k = 5 | 29	L = 1 to 14

### 4.3 Experimental result

The actual performance of an algorithm in real life is greatly affected by not only how it is designed but also how it is implemented, the data structure, the programming language, the compiler, and even the hardware. So, to make sure that the comparison is fair, every algorithm must be set up in a similar way. The goal of our experiment was to see how well each algorithm worked in different constrained settings. We wanted to see how well they could adapt and survive, not how quickly or efficiently they could use space or time. That is why we implemented Wall-L Sort, Merge Sort, Quick Sort, IntroSort, and TimSort, etc, using fully theoretical, raw Python code without any additional support functions for handling specific situations. We simply observed how each algorithm naturally behaved and survived in different environments, which is the main objective of this paper.

#### 4.3.1 High computing environment.

We conducted experiments on the proposed Wall-L Sort algorithm alongside several well-established existing algorithms, including Timsort, Introsort, Merge Sort, and Quick Sort. The high computing environment refers to a setting without stack or memory limitations. To simulate such conditions, all experiments were performed using random data in Google Colab using Python 3 on a standard CPU runtime. In this environment, Timsort was implemented in its iterative form, as it is inherently non-recursive, while all other algorithms were implemented using their recursive versions.

**Fig 4:**
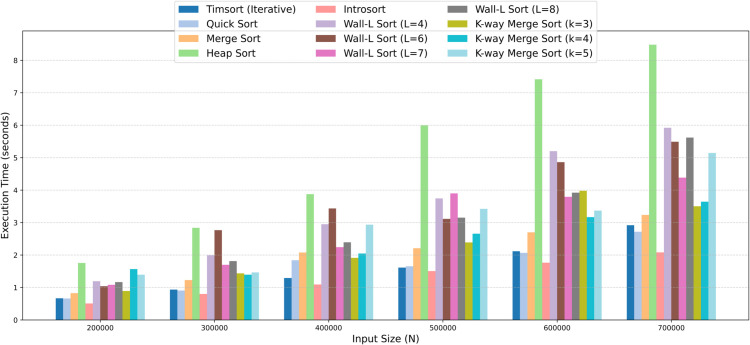
The grouped bar chart illustrates how the execution time scales with input size (N) for each algorithm in a high computing environment.

In the high computing environment without stack memory constraints, all algorithms were successfully executed and produced correct outputs. The results indicate that, along with the existing algorithms, Wall-L Merge Sort also provides consistent and alignable performance. For example, the Table 4 and Fig 4 show, at N=400,000, Wall-L Sort with *L* = 7 completed in 2.2439 seconds, closely comparable to Merge Sort (2.0784 s) and faster than Heap Sort (3.8772 s). At N=700,000, it maintained stability with 4.3852 seconds, showing adaptability and balanced growth similar to other recursive algorithms. These results show that Wall-L Merge Sort is practically adaptable to high-computing environments and works just as well as other well-known sorting algorithms.

**Table 4:** Experimental results of approximate execution times in seconds of different sorting algorithms under a high computing environment.

Algorithm	N=200K	N=300K	N=400K	N=500K	N=600K	N=700K
Timsort (Iterative)	0.6672	0.9347	1.2941	1.6140	2.1182	2.9189
Quick Sort	0.6608	0.9064	1.8391	1.6520	2.0693	2.7158
Merge Sort	0.8254	1.2294	2.0784	2.2096	2.7011	3.2360
Heap Sort	1.7540	2.8384	3.8772	5.9942	7.4141	8.4814
Introsort	0.5088	0.8019	1.0924	1.5043	1.7638	2.0828
Wall-L Sort (L=4)	1.1940	1.9912	2.9497	3.7440	5.1998	5.9190
Wall-L Sort (L=6)	1.0406	2.7663	3.4350	3.1131	4.8591	5.4899
Wall-L Sort (L=7)	1.0821	1.6992	2.2439	3.8985	3.7938	4.3852
Wall-L Sort (L=8)	1.1646	1.8138	2.3932	3.1512	3.9211	5.6179
K-way Merge Sort (k=3)	0.8925	1.4357	1.9135	2.3874	3.9792	3.5034
K-way Merge Sort (k=4)	1.5676	1.3936	2.0469	2.6565	3.1675	3.6432
K-way Merge Sort (k=5)	1.3961	1.4631	2.9368	3.4211	3.3700	4.5944

#### 4.3.2 Stack depth limiting environment.

We set up a simulated stack-limiting environment in Google Colab for this experiment on purpose. We created an artificial recursion depth control environment using auxiliary code. We just wanted to see which recursive sorting algorithms could naturally survive and work well under these conditions. We didn’t test Timsort because it is inherently iterative, which means we could focus only on the behavior of truly recursive algorithms.

The Table 5 shows that the traditional recursive algorithms, including Quick Sort, Merge Sort, Heap Sort, and Introsort, completely failed with *RecursionError* upon stack depth limit falling below 100. Rather, the proposed Wall-L Sort has shown high adaptability, properly functioning in even rigorous constraints stack limits down to 8 for most *L* values. This means that the wall-L has self-adaptability without jumping over the recursion depth. In a similar fashion, K-way merge sort with large k, for instance, *k* = 10 or *k* = 27, also survived in very small stack limits due to per-level reduced recursive branching, but high k values consume more execution time than the wall sort. The results presented on the Table 5 are evidence that Wall-L Sorts retains robustness, stability, and correctness when other recursive algorithms crash, positioning it as viable and feasible when considering the constraint computing environments such as stack limitation.

**Table 5:** Experimental results show execution time and survivability of recursive sorting algorithms under artificial stack-depth limiting environments (N=500,000). The sign × indicates a recursion error, and ✓ indicates successful execution.

Algorithm	Limit = 100	Limit = 15	Limit = 12	Limit = 8	Limit = 5
Quick Sort	1.6913 (✓)	0.0794 (×)	0.2783 (×)	0.0915 (×)	0.0354 (×)
Merge Sort	2.2154 (✓)	0.0023 (×)	0.0031 (×)	0.0024 (×)	0.0025 (×)
Heap Sort (Recursive Heapify)	6.2646 (✓)	0.3041 (×)	0.5545 (×)	0.3036 (×)	0.2488 (×)
Introsort	1.4402 (✓)	0.0630 (×)	0.1727 (×)	0.0642 (×)	0.0341 (×)
Wall-L Sort (L=4)	3.7669 (✓)	3.7676 (✓)	3.7944 (✓)	4.4320 (✓)	3.7624 (✓)
Wall-L Sort (L=6)	2.9922 (✓)	3.1245 (✓)	3.0911 (✓)	3.2983 (✓)	0.0001 (×)
Wall-L Sort (L=7)	4.0943 (✓)	3.9537 (✓)	2.9958 (✓)	3.0546 (✓)	0.0001 (×)
Wall-L Sort (L=8)	3.1439 (✓)	3.1858 (✓)	4.3166 (✓)	0.0001 (×)	0.0001 (×)
K-way Merge Sort (k=3)	2.3931 (✓)	2.3631 (✓)	0.0015 (×)	0.0013 (×)	0.0013 (×)
K-way Merge Sort (k=4)	2.7190 (✓)	2.7419 (✓)	2.7437 (✓)	0.0010 (×)	0.0009 (×)
K-way Merge Sort (k=5)	3.7689 (✓)	3.8571 (✓)	2.7024 (✓)	0.0007 (×)	0.0008 (×)
K-way Merge Sort (k=10)	3.3359 (✓)	3.3507 (✓)	3.8155 (✓)	3.3343 (✓)	0.0003 (×)
K-way Merge Sort (k=27)	5.2620 (✓)	5.2156 (✓)	5.7168 (✓)	6.3785 (✓)	6.5249 (✓)

#### 4.3.3 Stack limiting parallel environment.

We also implemented the algorithms in a stack-limited parallel environment. We considered algorithms implementing fully natural parallel scopes, i.e.,. Since Heap Sort can only parallelize a small portion of its heapify operations and Timsort and Introsort do not have a fully parallel scope, we considered algorithms that have full potential, namely: Quick Sort, Merge Sort, K-way Merge Sort, and Wall-L Sort. We then implemented each algorithm in its parallel form with theoretical alignment, meaning that the algorithm was modified only as needed to be parallel. We used Python’s concurrent.futures library to be able to exploit the parallel performance of the multi-core CPU that we were using in Google Colab. We wanted to see how the algorithm’s naturally behaved in the environment rather than to evaluate their performance. The results, similar to those shown in Table 4, indicate that under a high recursion limit, the algorithms operated efficiently within competitive execution times. However, under limited recursion depth, only Wall-L Sort and K-way Merge Sort naturally survived.

### 4.4 Discussion and comparative analysis

Iterative versions of algorithms can survive in stack-limiting environments. However, iterative versions are not fully aligned with parallel versions, because parallel environments require a divide-and-conquer nature that is easily achievable by recursive algorithms. In the modern era, a huge amount of data is processed continuously, and only parallel processing environments can provide results within a shorter time. The modern era is also the age of the Internet of Things (IoT), known as the Fourth Industrial Revolution. For the efficient construction of IoT networks in various sectors, embedded devices are essential. Embedded devices have limited stack memory and low processing speed to maintain energy efficiency and reduce cost. Therefore, the parallel environment in such embedded systems is subject to stack limitations. Surviving algorithms in this situation are crucial for performance robustness and meeting modern computational demands. It also serves as a good evaluation metric for analyzing which algorithm is best suited for a particular situation.

This paper analyzes this aspect by experimenting with existing algorithms in different environments to find naturally adaptable algorithms for any condition. The theoretical and experimental analysis shows that Timsort, which is used as Python’s default sorting algorithm, is fast and reliable but inherently iterative, giving it a very limited scope for parallelism. Introsort is also a powerful and fast algorithm, but our experiment shows that it fails in a stack-limited environment. Although Heap Sort has a theoretical complexity of O(nlogn), in practice it is comparatively slower due to cache inefficiency. Our experiment also shows that Heap Sort fails in a stack-limiting environment. Quick Sort is one of the fastest algorithms, but our experiment indicates that it becomes unreliable and fails under stack limitations. Merge Sort is a faster and stable algorithm, but it also suffers from stack depth issues. Our experiment shows that only the proposed Wall-L Sort and K-way Merge Sort naturally perform well in all environments.

The main disadvantage of K-way Merge Sort is that it does not have a stable recursion depth. The theoretical depth of K-way Merge Sort is $\log_{k}n$, which continuously changes based on *k* and *N*. If a parallel computing environment can tolerate a maximum recursion depth *D*, then Wall-L Sort can simply set *L* = *D*. It can then handle any data size *N* with a stable time complexity of $n\;n^{\frac{1}{D+1}}$. On the other hand, K-way Merge Sort requires continuous recalculation of *k* based on the sample size *N*. Our experiments also show that K-way Merge Sort can handle limiting depths by increasing the value of *k*, but increasing *k* also increases the execution time compared to Wall-L Sort. This is one of the disadvantages of handling large data in stack-limiting parallel environments such as embedded devices. In contrast, Wall-L Sort can handle any data size with stable and lower time complexity compared to K-way Merge Sort in stack-limiting environments. Also, Wall-L Merge Sort has a natural switching mechanism to a stable quadratic sort, such as Insertion Sort, which allows it to handle stack failure situations, a capability that is not present in K-way Merge Sort. Based on our theoretical and experimental results, Table 6 shows the comparison between established existing algorithms and the proposed Wall-L Sort.

**Table 6:** Feature-wise comparison of Wall-*L* sorting with classical sorting algorithms.

Feature	Wall-*L*	Merge	Quick	Heap	K-Way	Timsort	Introsort
Complexity Transition	✓	×	×	×	×	×	×
Stack-Control Execution	✓	×	×	×	✓	Iterative	×
Performance Robustness	✓	×	×	×	×	Partial	Partial
Platform Adaptability	✓	Limited	Limited	Limited	✓	Partial	Limited
Input-Aware Behavior	✓	×	×	×	×	✓	Partial
Asymptotic Efficiency	Optimal	Optimal	Optimal	Optimal	Optimal	Optimal	Optimal
Cache Miss Awareness	High	High	Low	Low	Medium	Medium	Medium
Parallelization Scope	High(Block-wise)	High	High	Medium	High	Low	Medium

## 5 Conclusion

Algorithms are typically evaluated based on time complexity or space complexity in the literature, but this paper presents a new insight into assessing algorithms through diverse constraint environment adaptability, which is truly needed in modern times. The paper assessed several established algorithms in different constrained environments based on their pure theoretical nature. The theoretical and experimental results show that each algorithm has specific advantages in certain conditions, but most algorithms fail in stack-constrained parallelism environments where recursive forms are necessary. Although it is possible to handle these algorithms using supporting functions in such situations, this approach restricts their theoretical predictability. We observed that only the proposed Wall-L and K-way Merge Sort have a natural scope to perform well in all situations. The paper also analyzed the advantages of Wall-L Sort over K-way Merge Sort. From the theoretical section, we observed that Wall-L uses a different merging strategy, where the main objective is to design a theoretically predictable depth-controlled algorithm. Asymptotically, we found that the Wall-L recursion depth is $\frac{\log n}{\log\log n}$, which is lower than logn, while achieving optimal time complexity of O(nlogn). Wall-L can theoretically run at any allowable depth independent of *N*, providing significant advantages in stack memory-constrained parallel environments. We also proved that Wall-L has optimal time complexity O(nlogn), efficient cache complexity $O\left(\frac{n}{P}\log\frac{n}{Z}\right)$ similar to pure Merge Sort, and natural parallelism with theoretical stack control execution. The limitation of Wall-L is that it is not best for every specific case. Although it has asymptotically optimal time complexity, for finite *L*, its execution time may be slightly affected due to the naturally reduced depth. In high-performance environments, we observed that despite of this algorithm provides competitive execution times compared to other established algorithms, it takes slightly more time than Timsort and Introsort. Therefore, Wall-L Sort is competitive but not the fastest among existing algorithms. However, it stands out for its algorithmic survivability across different environments, providing stable and reliable theoretical performance alignment. All theoretical and experimental results indicate that Wall-L Merge Sort can be considered the most reliable algorithm for stack-limited parallelism environments, such as embedded devices, which is a modern requirement. This also implies that the algorithm is flexible enough to work in a variety of computing environments. The Timsort, Introsort, quick sort, and merge sort are suitable for high computing environments where highly efficient processing time is more important, such as high-traffic server sites. On the other hand, Wall-L sort is suitable for a dynamic constraint environment where reliability, correctness, and predictability are also more important with considerable processing time, such as embedded device used to process data for research purpose. Our future work will concentrate on creating a completely optimized Wall-L Sort implementation to guarantee the best feasible performance on a variety of platforms. Finally, by presenting the Wall-L Sort and encouraging the use of assessment algorithms under a greater range of environmental constraints, we hope that our current analysis contributes to the literature.

## Supporting information

S1 File Source code and execution environment used to generate all experimental results in this study. (PDF)
