# Supplementary material for: Wall-L merge sort: A tunable and adaptive sorting algorithm for diverse computing environments
Source: PLoS One. 2026 Feb 2;21(2):e0341993. doi: 10.1371/journal.pone.0341993 (PMC12863692; doi:10.1371/journal.pone.0341993)
Supplement: S1 File — (PDF) [file pone.0341993.s001.pdf]

Supporting Information

S1 File. Source code and execution environment for reproducing the experimental results.

Execution Environment

All experiments were conducted using Python 3 in Google Colab. The operating system was Linux (Ubuntu-based Colab environment). The hardware environment consisted of multicore Intel Xeon CPUs with approximately 12 GB RAM. No GPU acceleration was used. Execution times may vary slightly due to dynamic resource allocation in Colab.

How to Run

1. Upload the provided Python scripts to a Python 3 environment.
2. Install required libraries: numpy, time, math, concurrent.futures.
3. Run the non-parallel version using: python Experiment1.py
4. Run the parallel version using: python Experiment2.py
5. The programs print execution time, recursion behavior, and correctness status to the console.

Appendix A: Non-parallel Implementation (Experiment1.py)

```
# -*- coding: utf-8 -*-
"""

# Experimental Setup Description

This Colab implementation creates an artificial stack-limiting
environment to simulate algorithm behavior under constrained
recursion depth. The goal is to evaluate how algorithms adapt in
both high-computing and stack-limited environments.

All algorithms are implemented in their pure theoretical form
without supporting code, allowing natural adaptability to be
observed. The environment is built in Google Colab for easy
reproducibility and verification.

**Two experimental portions are used in main function:**

1.   High-computing environment: large stack depth (e.g., 500)
with varying input size N.
2.   Stack-limiting environment: fixed N=500,000 with varying
stack depth limits.

The results and execution times presented in the paper are taken
```

```
directly from these runs. Due to dynamic CPU allocation in Colab,
execution times may slightly vary between runs, but overall
trends remain consistent.
"""

import numpy as np
import time
import math
import sys

LOWER_LIMIT = 1000
TEST_N = 3500000

# ---------------------- ARTIFICIAL STACK LIMIT ----------------
-----
current_depth = 0
max_depth = 30  # dynamically changed in test

def enter_depth():
    global current_depth
    current_depth += 1
    if current_depth > max_depth:
        raise RecursionError(f"Artificial recursion depth
exceeded: {current_depth}")

def exit_depth():
    global current_depth
    current_depth -= 1


# ---------------------- INSERTION SORT ----------------------
def insertion_sort(arr, left, right):
    for i in range(left + 1, right):
        key = arr[i]
        j = i - 1
        while j >= left and arr[j] > key:
            arr[j + 1] = arr[j]
            j -= 1
        arr[j + 1] = key


# ---------------------- WALL-L SORT ----------------------

#Calculating the block size using formula (nL/2)^(L/(L+1))
def wall_distance_calculate(layer_no, N, L):
```

```
    base = float(N)
    if layer_no > L:
        return base
    i = L
    while i >= layer_no:
        index = float(i) / (i + 1)
        base = pow(i * base / 2, index)
        i -= 1
    return base

def storing_walls(N, L):
    walls = [0] * (L + 1)
    for i in range(1, L + 1):
        walls[i] = int(wall_distance_calculate(i, N, L)) + 1
    return walls

def merge_blocks(arr, left, mid, right):
    Larr = arr[left:mid]
    Rarr = arr[mid:right]
    i = j = 0
    k = left
    while i < len(Larr) and j < len(Rarr):
        if Larr[i] <= Rarr[j]:
            arr[k] = Larr[i]
            i += 1
        else:
            arr[k] = Rarr[j]
            j += 1
        k += 1
    while i < len(Larr):
        arr[k] = Larr[i]
        i += 1
        k += 1
    while j < len(Rarr):
        arr[k] = Rarr[j]
        j += 1
        k += 1

def wall_sort_recursive(arr, left, right, layer, walls):
    enter_depth()
    try:
        if layer < 1:
            insertion_sort(arr, left, right)
            return
```

```
        start = left
        boundaries = [left]
        while True:
            next_pos = start + walls[layer]
            if next_pos <= start or next_pos >= right:
                break
            boundaries.append(next_pos)
            start = next_pos
        boundaries.append(right)

        for i in range(len(boundaries) - 1):
            l, r = boundaries[i], boundaries[i + 1]
            wall_sort_recursive(arr, l, r, layer - 1, walls)


        left_b = boundaries[0]
        for i in range(1, len(boundaries)):
            mid_b = boundaries[i - 1]
            right_b = boundaries[i]
            merge_blocks(arr, left_b, mid_b, right_b)
    finally:
        exit_depth()

def wall_sort(arr, L=6):
    N = len(arr)
    walls = storing_walls(N, L)
    wall_sort_recursive(arr, 0, N, L, walls)


# ---------------------- MERGE SORT ----------------------
def merge(left, right):
    result = []
    i = j = 0
    while i < len(left) and j < len(right):
        if left[i] <= right[j]:
            result.append(left[i])
            i += 1
        else:
            result.append(right[j])
            j += 1
    result.extend(left[i:])
    result.extend(right[j:])
    return result

def merge_sort(arr):
```

```
    enter_depth()
    try:
        if len(arr) <= 1:
            return arr
        mid = len(arr)//2
        left = merge_sort(arr[:mid])
        right = merge_sort(arr[mid:])
        return merge(left, right)
    finally:
        exit_depth()


# ---------------------- QUICK SORT ----------------------
def quick_sort(arr, low=0, high=None):
    enter_depth()
    try:
        if high is None:
            high = len(arr) - 1
        if low < high:
            p = partition(arr, low, high)
            quick_sort(arr, low, p - 1)
            quick_sort(arr, p + 1, high)
        return arr
    finally:
        exit_depth()

def partition(arr, low, high):
    pivot = arr[high]
    i = low - 1
    for j in range(low, high):
        if arr[j] <= pivot:
            i += 1
            arr[i], arr[j] = arr[j], arr[i]
    arr[i + 1], arr[high] = arr[high], arr[i + 1]
    return i + 1


# ---------------------- HEAP SORT ----------------------
def heapify(arr, n, i):
    enter_depth()  # simulate entering recursion frame
    try:
        largest = i
        l = 2*i + 1
        r = 2*i + 2
        if l < n and arr[l] > arr[largest]:
```

```
            largest = l
        if r < n and arr[r] > arr[largest]:
            largest = r
        if largest != i:
            arr[i], arr[largest] = arr[largest], arr[i]
            heapify(arr, n, largest)  # recursive call also
checked
    finally:
        exit_depth()


def heap_sort(arr):
    n = len(arr)
    for i in range(n//2 -1, -1, -1):
        heapify(arr, n, i)
    for i in range(n-1, 0, -1):
        arr[0], arr[i] = arr[i], arr[0]
        heapify(arr, i, 0)


# ---------------------- INTROSORT ----------------------
def introsort_helper(arr, start, end, maxdepth):
    enter_depth()
    try:
        size = end - start
        if size <= 1:
            return
        elif maxdepth == 0:
            temp = arr[start:end]
            heap_sort(temp)
            arr[start:end] = temp
        else:
            pivot = arr[start + size // 2]
            left = start
            right = end - 1
            while left <= right:
                while arr[left] < pivot:
                    left += 1
                while arr[right] > pivot:
                    right -= 1
                if left <= right:
                    arr[left], arr[right] = arr[right], arr[left]
                    left += 1
                    right -= 1
            introsort_helper(arr, start, right + 1, maxdepth - 1)
```

```
            introsort_helper(arr, left, end, maxdepth - 1)
    finally:
        exit_depth()

def introsort(arr):
    if len(arr) == 0:
        return
    maxdepth = int(2 * math.log2(len(arr)))
    introsort_helper(arr, 0, len(arr), maxdepth)


# ---------------------- TIMSORT ----------------------
def timsort(arr):
    min_run = 32
    n = len(arr)
    for start in range(0, n, min_run):
        end = min(start+min_run, n)
        arr[start:end] = sorted(arr[start:end])
    size = min_run
    while size < n:
        for start in range(0, n, size*2):
            mid = start + size
            end = min(start+size*2, n)
            if mid < end:
                merged_result = merge(arr[start:mid],
arr[mid:end])
                arr[start:end] = merged_result
        size *= 2


# ----------------------K-WAY MERGE SORT ----------------------
def k_way_merge_sort(arr, k=6):
    enter_depth()
    try:
        n = len(arr)
        if n <= 1 or k <= 1:
            return arr
        base = n // k
        remainder = n % k
        parts = []
        start = 0
        for i in range(k):
            size = base + (1 if i < remainder else 0)
            end = start + size
            parts.append(k_way_merge_sort(arr[start:end], k))
```

```
            start = end
        merged = []
        first = True
        for part in parts:
            if not part:
                continue
            if first:
                merged = part
                first = False
            else:
                merged = merge(merged, part)
        return merged if not first else []
    finally:
        exit_depth()


# ---------------------- STACK LIMIT TEST ----------------------
def run_stack_limit_test(N=TEST_N, limit=10, wall_L=[8],k=[3]):
    global max_depth, current_depth
    print(f"---Artificial Stack Test Environment---")
    print(f"Artificial Recursion Limit: {limit}")
    print(f"Array Size (N): {N}\n")

    max_depth = limit
    current_depth = 0

    data = np.random.randint(0, 10000000, N)
    sorted_ref = sorted(data.tolist()) # To check correctly
sorted data or not

    algorithms = {
        "Timsort (Iterative)": lambda d: timsort(d),
        "Quick Sort (Recursive)": lambda d: quick_sort(d),
        "Merge Sort (Recursive)": lambda d: merge_sort(d),
        "Heap Sort (recursive heapify)": lambda d: heap_sort(d),
        "Introsort (Recursive)": lambda d: introsort(d),

    }

    # Add Wall-L Sort iterations
    for wall_L in wall_L:
        name = f"Wall-L Sort recursive (L={wall_L})"
        algorithms[name] = lambda d, l=wall_L: wall_sort(d, l)

    # Add K-way Merge Sort iterations
```

```
    for k in k:
        name = f"K-way Merge Sort recursive (k={k})"
        algorithms[name] = lambda d, kv=k: k_way_merge_sort(d,
kv)

    print("Algorithm                  | Time (sec) | Result")
    print("--------------------------|------------|--------------
-------------")

    for name, func in algorithms.items():
        arr = data.copy().tolist()
        result = None
        elapsed = 0.0
        status = "PASSED & Correctly Sorted"
        current_depth = 0

        try:
            start = time.time()
            result = func(arr)
            elapsed = time.time() - start
            if result is not None:
                arr = result
            if arr != sorted_ref:
                status = "PASSED but INCORRECT"
        except RecursionError:
            elapsed = time.time() - start
            status = "FAILED: RecursionError"
        except Exception as e:
            elapsed = time.time() - start
            status = f"FAILED: {type(e).__name__}"

        print(f"{name:26s} | {elapsed:10.4f} | {status}")

    print("\nArtificial stack limit restored to normal
simulation.")


# ---------------------- MAIN ----------------------
if __name__ == '__main__':

    print(" ---------High Computing Environment No stack memory
limit-------------")
    print()
    N=[100000,200000,300000,400000,500000,600000,700000]
    for i in N:
```

```
      run_stack_limit_test(N=i,
limit=500,wall_L=[4,6,7,8],k=[3,4,5])


    print("-------------Stack Limiting Environment---------------
-------")
    print()
    stack_limit=[100,15,12,8,5]
    for i in stack_limit:
      run_stack_limit_test(N=500000,
limit=i,wall_L=[4,6,7,8],k=[3,4,5,10,27])
```

Appendix B: Parallel Implementation (Experiment2.py)

```
# -*- coding: utf-8 -*-
"""

# Parallel Implementation Description

Each algorithm was also implemented in a parallel version using
the concurrent.futures library to enable multicore CPU execution.
The implementations remain theoretically aligned with their
original structures, with minimal modification made only to
support parallel execution.

The goal of this experiment was to observe the adaptability of
the parallel versions in the artificial stack-limiting
environment, rather than to achieve the best possible parallel
performance.
"""

import numpy as np
import time
import concurrent.futures
import heapq
import copy
import sys
import math

TEST_N = 500000
MAX_WORKERS = 400


def insertion_sort(arr, left, right):
    for i in range(left + 1, right):
        key = arr[i]
```

```
        j = i - 1
        while j >= left and arr[j] > key:
            arr[j + 1] = arr[j]
            j -= 1
        arr[j + 1] = key


def merge_blocks(arr, left, mid, right):
    L = arr[left:mid]
    R = arr[mid:right]
    i = j = 0
    k = left
    while i < len(L) and j < len(R):
        if L[i] <= R[j]:
            arr[k] = L[i]; i += 1
        else:
            arr[k] = R[j]; j += 1
        k += 1
    while i < len(L):
        arr[k] = L[i]; i += 1; k += 1
    while j < len(R):
        arr[k] = R[j]; j += 1; k += 1


# ----------------- Wall-L Parallel Implementation --------------
---

def wall_distance_calculate(layer_no, N, L):
    base = float(N)
    if layer_no > L:
        return base
    i = L
    while i >= layer_no:
        index = float(i) / (i + 1)
        base = pow(i * base / 2, index)
        i -= 1
    return base

def storing_walls(N, L):
    walls = [0] * (L + 1)
    for i in range(1, L + 1):
        walls[i] = int(wall_distance_calculate(i, N, L)) + 1
    return walls

def wall_sort_recursive(arr, left, right, layer, walls, depth=0,
```

```
stack_limit=None):
    if stack_limit is not None and depth > stack_limit:
        raise RecursionError(f"Stack limit reached at depth
{depth} in Wall-L")

    if layer < 1:
        insertion_sort(arr, left, right)
        return

    start = left
    boundaries = [left]
    while True:
        next_pos = start + walls[layer]
        if next_pos <= start or next_pos >= right:
            break
        boundaries.append(next_pos)
        start = next_pos
    boundaries.append(right)

    futures = []
    with
concurrent.futures.ThreadPoolExecutor(max_workers=MAX_WORKERS) as
executor:
        for i in range(len(boundaries) - 1):
            l = boundaries[i]; r = boundaries[i+1]
            futures.append(executor.submit(wall_sort_recursive,
arr, l, r, layer-1, walls, depth+1, stack_limit))

        for f in futures:
            f.result()

    left_b = boundaries[0]
    for i in range(1, len(boundaries)):
        mid_b = boundaries[i-1]
        right_b = boundaries[i]
        merge_blocks(arr, left_b, mid_b, right_b)

def wall_sort_parallel(arr, L=6, stack_limit=None):
    N = len(arr)
    walls = storing_walls(N, L)
    wall_sort_recursive(arr, 0, N, L, walls, depth=0,
stack_limit=stack_limit)
    return arr
```

```
# ----------------- Quick Sort Parallel Implementation ----------
-------

def partition(arr, low, high):
    pivot = arr[high]
    i = low - 1
    for j in range(low, high):
        if arr[j] <= pivot:
            i += 1
            arr[i], arr[j] = arr[j], arr[i]
    arr[i+1], arr[high] = arr[high], arr[i+1]
    return i+1

def quick_sort_parallel(arr, low=0, high=None, depth=0,
max_parallel_depth=4, stack_limit=None):

    if high is None:
        high = len(arr) - 1

    if stack_limit is not None and depth > stack_limit:
        raise RecursionError(f"Stack limit reached at depth
{depth} in QuickSort")

    if low >= high:
        return

    pi = partition(arr, low, high)

    if depth < max_parallel_depth:
        with concurrent.futures.ThreadPoolExecutor(max_workers=2)
as executor:
            left_f = executor.submit(quick_sort_parallel, arr,
low, pi-1, depth+1, max_parallel_depth, stack_limit)
            right_f = executor.submit(quick_sort_parallel, arr,
pi+1, high, depth+1, max_parallel_depth, stack_limit)
            left_f.result()
            right_f.result()
    else:
        quick_sort_parallel(arr, low, pi-1, depth+1,
max_parallel_depth, stack_limit)
        quick_sort_parallel(arr, pi+1, high, depth+1,
max_parallel_depth, stack_limit)

    return arr
```

```
# ----------------- Merge Sort Parallel -----------------
def merge_sort_parallel(arr, left=0, right=None, depth=0,
max_parallel_depth=4, stack_limit=None):

    if right is None:
        right = len(arr)

    if stack_limit is not None and depth > stack_limit:
        raise RecursionError(f"Stack limit reached at depth
{depth} in MergeSort")

    if right - left <= 1:
        return

    mid = (left + right) // 2

    if depth < max_parallel_depth:
        with concurrent.futures.ThreadPoolExecutor(max_workers=2)
as executor:
            left_f = executor.submit(merge_sort_parallel, arr,
left, mid, depth+1, max_parallel_depth, stack_limit)
            right_f = executor.submit(merge_sort_parallel, arr,
mid, right, depth+1, max_parallel_depth, stack_limit)
            left_f.result()
            right_f.result()
    else:
        merge_sort_parallel(arr, left, mid, depth+1,
max_parallel_depth, stack_limit)
        merge_sort_parallel(arr, mid, right, depth+1,
max_parallel_depth, stack_limit)

    merge_blocks(arr, left, mid, right)
    return arr



# ==================== PARALLEL K-WAY MERGE SORT
====================


def k_way_merge_sort(arr, k=4, depth=0, max_parallel_depth=4,
stack_limit=None):
```

```
    """Returns the merged list, not in-place."""

    if stack_limit is not None and depth > stack_limit:
        raise RecursionError(f"Stack limit reached at depth
{depth} in K-way MergeSort")

    n = len(arr)
    if n <= 1:
        return arr

    chunk_size = (n + k - 1) // k
    subarrays = [arr[i:i + chunk_size] for i in range(0, n,
chunk_size)]

    if depth < max_parallel_depth and len(subarrays) > 1:
        with
concurrent.futures.ThreadPoolExecutor(max_workers=min(k,
len(subarrays), MAX_WORKERS)) as executor:
            futures = [
                executor.submit(k_way_merge_sort, sub, k, depth +
1, max_parallel_depth, stack_limit)
                for sub in subarrays
            ]
            sorted_parts = [f.result() for f in futures]
    else:
        sorted_parts = [
            k_way_merge_sort(sub, k, depth + 1,
max_parallel_depth, stack_limit)
            for sub in subarrays
        ]

    merged = []
    heap = []
    for i, part in enumerate(sorted_parts):
        if part:
            heapq.heappush(heap, (part[0], i, 0))
    while heap:
        val, i, j = heapq.heappop(heap)
        merged.append(val)
        if j + 1 < len(sorted_parts[i]):
            heapq.heappush(heap, (sorted_parts[i][j + 1], i, j +
1))

    return merged
```

```
# ---------------------- TEST CONTROL ----------------------

def run_stack_limit_test_theoretical(N=TEST_N, limit=10,
wall_L_values=[2, 5, 6, 7], k_values=[2, 3, 4, 5]):




    print(f"--- Theoretical Parallel Stack Limit Test ---")
    print(f"Array Size (N): {N} | Artificial Recursion Limit:
{limit}\n")

    #Random Data
    data = np.random.randint(0, 10000000, N)
    # Phython defult sort for checking correctness of each
algorithm
    sorted_ref = sorted(data.tolist())

    algorithms = {}

    # 1. Quick Sort and Merge Sort
    algorithms[f"Quick Sort"] = lambda d: quick_sort_parallel(d,
stack_limit=limit)
    algorithms[f"Merge Sort"] = lambda d: merge_sort_parallel(d,
stack_limit=limit)

    # 2. Wall-L Sort iterations

    for L_val in wall_L_values:
        name = f"Wall-L Sort (L={L_val})"
        algorithms[name] = lambda d, l=L_val:
wall_sort_parallel(d, L=l, stack_limit=limit)

    # 3. K-way Merge Sort iterations
    for k_val in k_values:
        name = f"K-way Merge (k={k_val})"
        # K-way merge returns a new list, so it handles the array
copy internally
        algorithms[name] = lambda d, kv=k_val:
k_way_merge_sort(copy.copy(d), k=kv, stack_limit=limit)


    print(f"{'Algorithm':25s} | {'Time (sec)':10s} |
{'Result':30s} | Depth vs Limit")
```

```
    print("-------------------------|------------|--------------
------------------|--------------")

    for name, func in algorithms.items():
        if "K-way Merge" not in name:
            arr = data.copy().tolist()
        else:
            arr = data.copy().tolist()

        result = None
        elapsed = 0.0
        status = "PASSED & Correctly Sorted"

        try:
            start = time.time()
            # Call the function
            result = func(arr)
            elapsed = time.time() - start

            # Check correctness
            final_arr = arr if result is None or "K-way Merge"
not in name else result
            if final_arr != sorted_ref:
                status = "PASSED but INCORRECT"

            # Since depth is not captured globally, we report the
theoretical depth based on log2(N)
            if N > 1:
                depth_check = f"{int(math.log2(N)):<3d} >
{limit}"
            else:
                depth_check = f"N/A"

        except RecursionError:
            elapsed = time.time() - start
            status = "FAILED: RecursionError"
            depth_check = f"FAILED > {limit}"
        except Exception as e:
            elapsed = time.time() - start
            status = f"FAILED: {type(e).__name__}"
            depth_check = f"ERROR"

        # Wall-L and K-way have variable depth; Quick/Merge is
log2(N)
        if not status.startswith("FAILED"):
```

```
            if name.startswith("Wall-L"):
               depth_check = f"L={L_val} vs {limit}"
            elif name.startswith("K-way"):
               depth_check = f"k={k_val} vs {limit}"


        print(f"{name:25s} | {elapsed:10.4f} | {status:30s} |
{depth_check}")


# ---------------------- MAIN EXECUTION ----------------------
if __name__ == '__main__':

    WALL_L_VALUES = [1,2,3,4,7,8]
    K_VALUES = [3,4,5,10]


print("\n=========================================================="
)
    print("Test: Low Stack Limit Prallel Environment")

print("==========================================================")
    stack_limit=[100,15,12,8,5]
    for i in stack_limit:
      run_stack_limit_test_theoretical(
        N=70000,
        limit=i, # Low limit to force RecursionError
        wall_L_values=WALL_L_VALUES,
        k_values=K_VALUES
    )
```
